# Chlortetracycline-Functionalized Silver Nanoparticles as a Colorimetric Probe for Aminoglycosides: Ultrasensitive Determination of Kanamycin and Streptomycin

**DOI:** 10.3390/nano10050997

**Published:** 2020-05-22

**Authors:** Ganesh Dattatraya Saratale, Rijuta Ganesh Saratale, Gajanan Ghodake, Surendra Shinde, Dae-Young Kim, Abdullah A. Alyousef, Mohammed Arshad, Asad Syed, Deepak Pant, Han-Seung Shin

**Affiliations:** 1 Department of Food Science and Biotechnology, Dongguk University-Seoul, 32 Dongguk-ro, Ilsandong-gu, Goyang-si, Gyeonggi-do 10326, Korea; gdsaratale@dongguk.edu; 2 Research Institute of Biotechnology and Medical Converged Science, Dongguk University-Seoul, 32 Dongguk-ro, Ilsandong-gu, Goyang-si, Gyeonggi-do 10326, Korea; rijutaganesh@gmail.com; 3 Department of Biological and Environmental Science, Dongguk University-Seoul, 32 Dongguk-ro, Ilsandong-gu, Goyang-si, Gyeonggi-do 10326, Korea; ghodakegs@gmail.com (G.G.); shindesurendra9@gmail.com (S.S.); sbpkim@dongguk.edu (D.-Y.K.); 4 Microbiology Research Group, Department of Clinical Laboratory Sciences, College of Applied Medical Sciences, King Saud University, P.O. Box 10219, Riyadh 11433, Saudi Arabia; abalyousef@ksu.edu.sa (A.A.A.); marshad@ksu.edu.sa (M.A.); 5 Department of Botany and Microbiology, College of Science, King Saud University, P.O. Box 2455, Riyadh 11451, Saudi Arabia; asadsayyed@gmail.com; 6 Separation and Conversion Technology, Flemish Institute for Technological Research (VITO), Boeretang 200, 2400 Mol, Belgium; deepak.pant@vito.be

**Keywords:** chlorotetracycline antibiotics, silver nanoparticles, ultrasensitive detection, aminoglycoside antibiotics, water samples, picomolar level sensitivity

## Abstract

Aminoglycosides (AMGs) have been extensively used to treat infectious diseases caused by Gram-negative bacteria in livestock and humans. A selective and sensitive colorimetric probe for the determination of streptomycin and kanamycin was proposed based on chlortetracycline-coated silver nanoparticles (AgNPs–CTC) as the sensing element. Almost all of the tested aminoglycoside antibiotics can rapidly induce the aggregation of AgNPs, along with a color change from yellow to orange/red. The selective detection of aminoglycoside antibiotics, including tobramycin, streptomycin, amikacin, gentamicin, neomycin, and kanamycin, with other types of antibiotics, can be achieved by ultraviolet (UV) spectroscopy. This developed colorimetric assay has ability to detect various AMGs using in-depth surface plasmon resonance (SPR) studies. With this determination of streptomycin and kanamycin was achieved at the picomolar level (pM) by using a UV–visible spectrophotometer. Under aqueous conditions, the linear range of the colorimetric sensor for streptomycin and kanamycin was 1000–1,1000 and 120–480 pM, respectively. The corresponding limit of detection was 2000 pM and 120 pM, respectively. Thus, the validated dual colorimetric and ratiometric method can find various analytical applications for the ultrasensitive and rapid detection of AMG antibiotics in water samples.

## 1. Introduction

Aminoglycosides (AMGs) are RNA-binding antibiotics sharing a common core structure named the streptamine ring. The mechanism of action involves binding to bacterial ribosomes, a vital role in the formation of nonsense peptides, finally leading to bacterial cell death [1]. Therefore, farmers tend to use AMG antibiotics to treat some bacterial infections in agricultural crops and livestock animals [2]. Recently, the clinical use of AMG antibiotics has been strictly defined because of its irreversible toxicity to vital organs, particularly ears and kidneys [3]. However, most AMG antibiotics are still often and extensively used in animal husbandry owing to their low-cost, causing potential residues in the water and food chain [2].

Test kits and other laboratory-based methods used for the detection of antibiotics are mainly based on microbial inhibition assays. Most of them, however, either cannot detect AMGs or have a much higher limit of detection (LOD) relative to the maximum residue limit [4]. Conventional liquid chromatography–tandem mass spectrometry (LC–MS/MS) offers an effective alternative to these methods [5] because it allows screening, confirmation, and validation of the target analyte. The disadvantages of this approach are expensive to operate when screening thousands of samples, requires highly qualified specialists, and cannot presently be used at remote sites [6]. Overcoming these issues requires the development of novel, rapid, and cost-effective methods suitable for laboratory and on-site screening (slaughterhouses or inspection posts) of AMGs.

Gold nanoparticles (AuNPs) have been used extensively for colorimetric detection of both inorganic and molecular targets in several settings, including water and food safety screening [7,8,9]. These include AuNPs functionalized with ssDNA aptamers, peptides, amino acids, and other biomolecules. In their simplest setting, these stabilizing agents can be coated onto the surface of metal nanoparticles (NPs). This surface passivation layer leads to target analyte-induced aggregation, resulting in a color change from red to blue for AuNPs and yellow to orange/red for silver nanoparticles (AgNPs), owing to surface plasmon resonance (SPR) coupling interactions among neighboring NPs [10]. These colorimetric changes can be easily observed by the eye, and enable target analyte quantification by spectroscopy. In high-throughput screening approaches, the preparation of functionalized AgNPs rather than AuNPs is more cost-effective because the cost of gold limits the affordability [11,12]. Despite many previous reports on the use of metal NPs for detecting antibacterial chemical compounds, the present study is the first report on the use of chlortetracycline-coated AgNPs (AgNPs–CTC) for probing the structures of AMG antibiotics.

## 2. Materials and Methods

### 2.1. Chemicals and Reagents

Tobramycin, doxycycline, and neomycin were purchased from Alfa Aesar (Haverhill, MA, USA). Oxytetracycline, amikacin, penicillin V, and metacycline were purchased from Cayman (Ann Arbor, MI, USA). Kanamycin, erythromycin, gentamycin, azithromycin, and clarithromycin were obtained from Tokyo Chemical Industry Co., Ltd (Tokyo, Japan). Silver nitrate (AgNO_3_), tetracycline, ampicillin, chlortetracycline, streptomycin, and penicillin G were procured from Sigma–Aldrich (St. Louis, MO, USA).

### 2.2. Synthesis of Nanoprobe

Synthesis of AgNPs–CTC was performed with minor modification of the previously reported method [13]. Briefly, dilute NaOH solution (0.1 mL, 1 mM) was added to 1 mM of CTC solution, and the total volume to 8 mL adjusted with nanopure water. The subsequent addition of AgNO_3_ (2 mL, 20 mM) under different temperature conditions (20, 40, 60, and 80 °C) was performed to improve the AgNPs synthesis. The colorless reaction mixture rapidly turned brown within a few minutes, showing a well-defined SPR band centered at 400 nm. Then, 0.2 mL aliquots of AgNPs collected under different temperature conditions were diluted to 0.8 mL with nanopure water. At 60 min after the AgNPs synthesis, the ultraviolet–visible (UV–vis) spectra of the samples were recorded in the range 300–800 nm using a spectrophotometer (Optizen 2120) equipped with an automatic rotary type 8-cell holder and 1 cm path length quartz cuvettes. The average of each triplicate measurement (*n* = 3) was used to observe the absorbance intensity at 400 nm and plotted against the temperature conditions to monitor the productivity. High-resolution transmission electron microscopy (HR-TEM) was performed on a Jeol JSM-2100F (Tokyo, Japan) to determine the size distribution and shape of the AgNPs. AgNPs were deposited onto glass and dried before analysis by X-ray photoelectron spectroscopy (XPS; Thermo Fisher Scientific, Loughborough, UK). A monochromatic Al K_α_ as an X-ray source was used to obtain the XPS data.

### 2.3. Purification of Silver Nanoparticles (AgNPs)

The solution of AgNPs was then purified by ultracentrifugation at 12,000 rpm for 15 min, redispersed in water to remove the excess organic groups from the surface of the AgNPs, and stored at room temperature. However, no color change was observed. The color of the reaction mixture remained yellow, suggesting the successful CTC conjugation and surface functionalization [14]. The lack of a color change was further confirmed by the UV–vis spectroscopy evaluation.

### 2.4. Selectivity of Nanoprobe

The selectivity of any analytical probe is important and investigated by noticing the change in color and red-shifts in the UV–vis spectra. The AMG antibiotics, including streptomycin, kanamycin, tobramycin, neomycin, amikacin, and gentamycin, were prepared by treating 0.1 mL of 1 µM stock solutions with the stock solution of AgNPs (50 μL) in 0.85 mL of water. For the application of the AgNPs probe as a colorimetric indicator specific to the AMGs, other antibiotics, including penicillin G, penicillin V, ampicillin, erythromycin, clarithromycin, azithromycin, tetracycline, chlortetracycline, oxytetracycline, metacycline, and doxycycline were tested under identical conditions. The UV–vis spectra and color of the AgNPs probe were recorded for all tested AMGs and other antibiotic samples after 20 min of reaction at ambient temperature (22–24 °C).

### 2.5. Sensitivity of AgNPs Probe

Quantification of the AMGs, both streptomycin (1000–11,000 pM) and kanamycin (120–480 pM) was performed in triplicate (*n* = 3) using a 30 μL AgNPs probe solution. The UV-vis spectra and ratiometric absorbance (A_540_/A_400_) were recorded for 20 min of treatment at ambient temperature. The LOD of the prepared AgNPs probe for both streptomycin and kanamycin was calculated, and the average value reported.

### 2.6. Effect of Ionic Strength on AgNPs Probe

The efficiency of the AgNPs probe was studied as a function of the ionic strength (5, 10, 15, 20, 25, 30, or 35 mM NaOH) using 30  μL aliquots of the AgNPs stock solution The AgNPs probe and NaOH solutions were thoroughly mixed in UV–vis cuvettes. After incubation for 5 min, the absorbance intensities were measured at 540 nm (*n* = 3). Streptomycin (0.18 mL, 50 nM) and kanamycin (0.035 mL, 12.5 nM) were then added, and the solutions mixed well. The AgNPs probe was allowed to interact with the target antibiotics for another 20 min at ambient temperature. The absorbance intensities of the dilute NaOH-treated AgNPs probe were again measured at 540 nm.

### 2.7. Stability of AgNPs Probe

A series of 30  μL-aliquots of the AgNPs stock solution was treated with 0.91, 0.87, 0.83, and 0.79 mL of water, and the initial absorbance intensities at 400 and 540 nm were measured. Afterward, the solutions were treated with 0.060, 0.10, 0.14, and 0.18 mL streptomycin (50 nM). The absorbance intensities at 400 and 540 nm were remeasured at 2 min intervals for up to 18 min.

## 3. Results and Discussion

### 3.1. Ultraviolet–Visible (UV–vis) Absorption Spectroscopy

**Figure 1:**
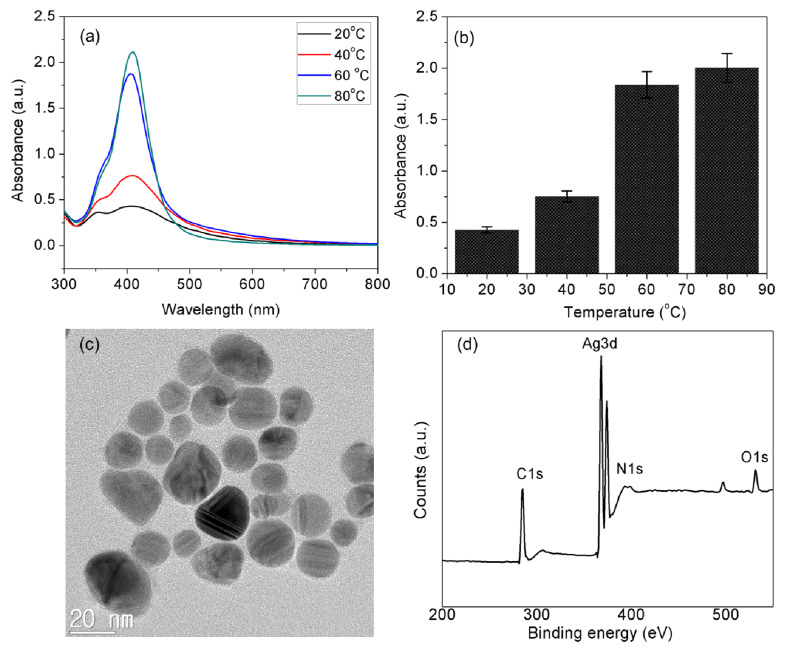
Effect of temperature conditions on (**a**) ultraviolet–visible (UV–vis) spectrum of AgNPs, (**b**) absorbance intensity of AgNPs at 400 nm, (**c**) high-resolution transmission electron microscopy (HR-TEM) analysis image at 20 nm and (**d**) X-ray photoelectron spectroscopy (XPS) spectrum of synthesized AgNPs.

Aqueous CTC was used as the reducing and stabilizing agent in the synthesis of the AgNPs. Dilute NaOH was used as a strong base to deprotonate the CTC molecules present in the reaction mixture. The deprotonated species of CTC show improved solubility in water and reduction of Ag^+^ ions. The addition of AgNO_3_ (2 mL, 20 mM) to the preheated basic CTC solution was performed under different temperature conditions (20, 40, 60, and 80 °C). The resultant formation of AgNPs was monitored for 60 min. The UV–vis spectra and absorbance results (Figure 1) are of diluted AgNPs (0.2 mL) mixed with 0.8 mL of nanopure water. The colorless reaction mixture turned brown within a few minutes, indicating the synthesis of AgNPs. An examination of the resultant dark yellow AgNPs by UV–vis absorption spectroscopy revealed an intense peak near 410 nm (Figure 1a). This color change in the reaction solution is caused by the reduction of Ag^+^ ions, as exhibited by the SPR peak at 410 nm. The sharp peak could be considered as the SPR band of AgNPs, which is dependent on the physicochemical properties of the AgNPs, including their particle size, shape, surface chemistry, medium, and ionic strength [15].

From the temperature response curve, the absorbance intensity of AgNPs at 410 nm increased gradually with the temperature and reached a maximum at 80 °C after 60 min (Figure 1b). However, the SPR peak position remained near 410 nm as the temperature increased. These results indicate that the synthesis of AgNPs could be completed at a rapid pace within this temperature range. The intensity of the SPR absorbance signal of AgNPs increased with the increase in temperature. Thus, the productivity of AgNPs was dependent on the temperature conditions; the higher the temperature, the more productivity. Although, temperature influences the formation and productivity of AgNPs [16], the role of the reaction temperature in the growth rate of AgNPs is yet to be well understood [17].

### 3.2. High-Resolution Transmission Electron Microscopy (HR-TEM) and X-ray Photoelectron Spectroscopy (XPS) Analysis

The AgNPs had an average size of about 14 nm and were monodispersed with mixed shapes but a predominance of spherical NPs, as illustrated in the representative electron micrograph (Figure 1c). It was evident that the AgNPs surface was encircled with CTC molecules. This organic layer is the CTC that was formed immediately after the synthesis of AgNPs by a conjugation reaction. The XPS spectrum of AgNPs shows Ag3d, O1s, C1s, and N1s, indicating the presence of both AgNPs and CTC molecules, and further confirms the conjugation (Figure 1d). The Ag3d core spectra consisted of two components, Ag3d_5/2_ and Ag 3d_3/2_, with binding energies of 368.1 and 374.2 eV, respectively. The Ag3d doublet was split by 6.1 eV, in agreement with the metallic silver and literature values [18,19]. As shown in Figure 1d, the C1s core spectra of the AgNPs had a single dominant peak with a binding energy of 284.5 eV, which is attributed to carbon atoms of phenyl rings. The appearance of the Cls and O1s species in the XPS spectra of the AgNPs indicates that the CTC molecules are appropriate to functionalize AgNPs. The O1s peak in the AgNPs–CTC has a single characteristic peak (531.3 eV) assignable to oxygen atoms of CTC molecules in C–OH/C–O–C. The N1s peak of AgNPs–CTC shows a single major component at 401.2 eV, which can be assigned to the amino groups (NH_2_) of CTC molecule functionalities. The CTC molecule is complex and shows different tautomeric forms, so it is difficult to determine the structure of CTC on the surface of NPs. However, according to the N1s spectra, CTC may conjugate to the AgNPs surface by an amide linkage because Ag^+^ ions have a high affinity for amide group-containing materials [20,21].

### 3.3. Selectivity

**Figure 2:**
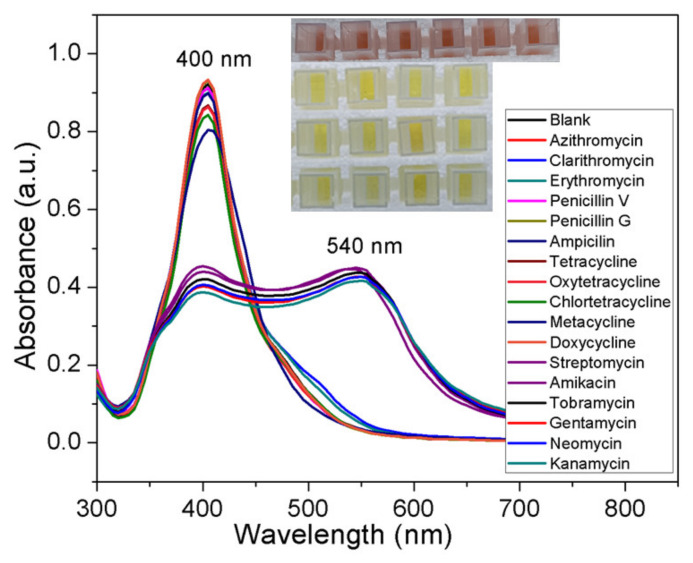
UV–vis spectra of chlortetracycline-coated silver nanoparticles after the addition of 100 nM of various antibiotics, including six types of aminoglycoside. One control with no antibiotics was included in the analysis.

To investigate the selectivity of the AgNPs probe, potentially interfering competitive antibiotics, such as streptomycin, kanamycin, tobramycin, neomycin, amikacin, and gentamycin of the AMG class, and other antibiotics, including penicillin G, penicillin V, ampicillin, erythromycin, clarithromycin, azithromycin, tetracycline, chlortetracycline, oxytetracycline, metacycline, and doxycycline, were performed under identical conditions. The corresponding visual appearance of each AgNPs probe sample is shown in the inset of Figure 2. The red solutions contained AMGs (first lane), whereas the other samples appeared yellow.

Figure 2 shows the changes in the UV–vis spectra of solutions of AgNPs probe with 100 nM of different antibiotics. These results demonstrate that antibiotics other than AMGs display a negligible colorimetric response and spectral shifts with the proposed AgNPs probe. Thus, the AgNPs probe displays exceptional selectivity toward AMGs owing to the strong affinity between the CTC ligand and AMGs.

### 3.4. Mechanism of Aminoglycosides (AMGs) Detection

**Figure 3:**
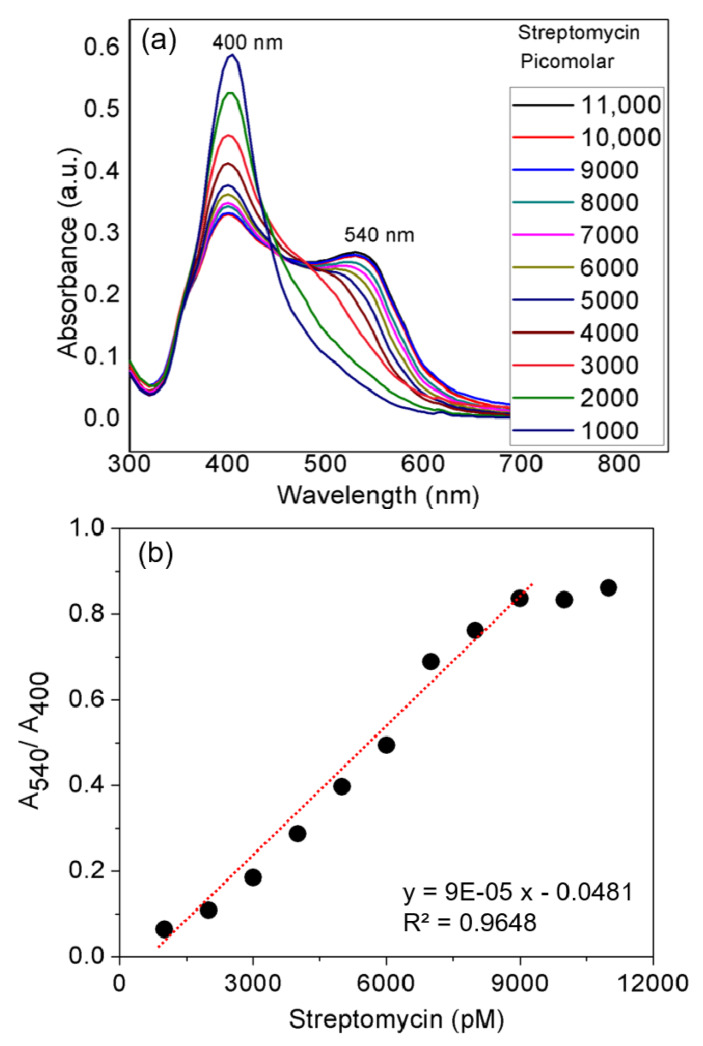
Streptomycin limit of detection analyzed using UV–vis spectrum and ratiometric results. Varying concentrations of streptomycin in water were added to chlortetracycline-coated silver nanoparticles in a final volume of 1 mL in the cuvette. (**a**) The UV–vis spectra obtained for each concentration of streptomycin and (**b**) the linear range observed for ratiometric measurement (A_540_/A_400_). The values represent the average of triplicates of each set.

Although effective in the treatment of various infectious diseases, AMG antibiotics have severe side effects, which limit their clinical use [22,23,24]. The abuse of AMG antibiotics in animal husbandry and the agricultural practices has led to their undesirable occurrence in food and the environment. AMG antibiotics are challenging to detect because they lack spectroscopic and electrochemical properties. Of the various analytical methods that have been proposed for these antibiotics, colorimetric or spectrophotometric detection using dyes [25] and metallic NPs, notably AuNPs and AgNPs [26,27,28,29], are the simplest methods. This method is based on the polycationic nature of the aminoglycoside, together with negatively-charged AgNPs. The interaction of opposite charges between negatively charged AgNPs and AMG antibiotics causes an aggregation-induced characteristic shift of the SPR band of the AgNPs probe in aqueous solutions. In the case of CTC-functionalized AgNPs, the antibiotic ligands bring the AgNPs into close contact, causing the SPR band to undergo a bathochromic shift in wavelength from 400 to 540 nm and a distinct visual color change from yellow to red (Figure 3a). The amine functional groups of the antibiotics act as a molecular linker, initiating the electrostatic coupling interactions among adjacent AgNPs and, ultimately, driving the formation of well-defined AgNP aggregates.

Most of the antibiotics that come under the class of AMGs carry a positive charge. Remarkably, however, not all of them cause aggregation of the AgNPs probe identically. When the ability of six different AMGs to cause the red-shift of the AgNPs probe was investigated under identical conditions, almost all the AMGs were distinguishable from the control sample. As can be seen in Figure 2, nearly all caused a noticeable red-shift in the SPR band, indicating that aggregation of the AgNPs probe can occur at the tested concentration. Therefore, it was difficult to determine whether the AMGs caused the same extent of bathochromic shift or if it varied depending on the number of amine groups. Streptomycin and kanamycin were selected as model AMG antibiotics for studying the broadening of the plasmon band and their quantification in the picomolar range.

### 3.5. Quantification of Model AMG Antibiotics Streptomycin and Kanamycin

Figure 3 shows the SPR data of CTC-coated AgNPs after the addition of increasing concentrations of streptomycin, from 1000 to 11,000 pM. As this was carried out using the UV–vis cuvettes and automated reader, analysis of all streptomycin concentrations (*n* = 3) was performed using a relatively small sample volume (1 mL) within 20 min. It is evident in Figure 3a that concentration-dependent aggregation of the AgNPs probe manifested itself as a decrease in the absorbance at 400 nm relative to the increase in the red-shifted peak at 540 nm. The ratio of these two A_540_/A_400_ absorbance wavelengths was calculated to provide a linear concentration plot between 1000 and 11,000 pM streptomycin (Figure 3b). Above 9000 pM, the AgNPs probe was fully aggregated at the fixed concentration of AgNPs used, and the linear range at the higher end resulted in an error caused by the lack of sufficient increase in the absorbance at 540 nm. Therefore, when the concentrations of streptomycin cause a response out of the linear range of the UV–vis absorbance, it would be difficult to analyze an exact concentration, so prior dilution is suggested. However, it is worth noting that high concentrations of AMG antibiotics can be easily detected by the naked eye. The AgNPs probe exhibited better analytical performance in terms of sensitivity than linearity. The LOD for the ratiometric results was about 2000 pM with a correlation coefficient (R^2^) of 0.964, for streptomycin. Thus, this proposed probe is a highly sensitive and colorimetric detection system for all kinds of AMG antibiotics at concentrations (picomolar) below the safe limit set for drinking water. The LOD of the AgNPs method is comparable to or lower than those of the reported methods for detecting streptomycin (Table 1).

**Figure 4:**
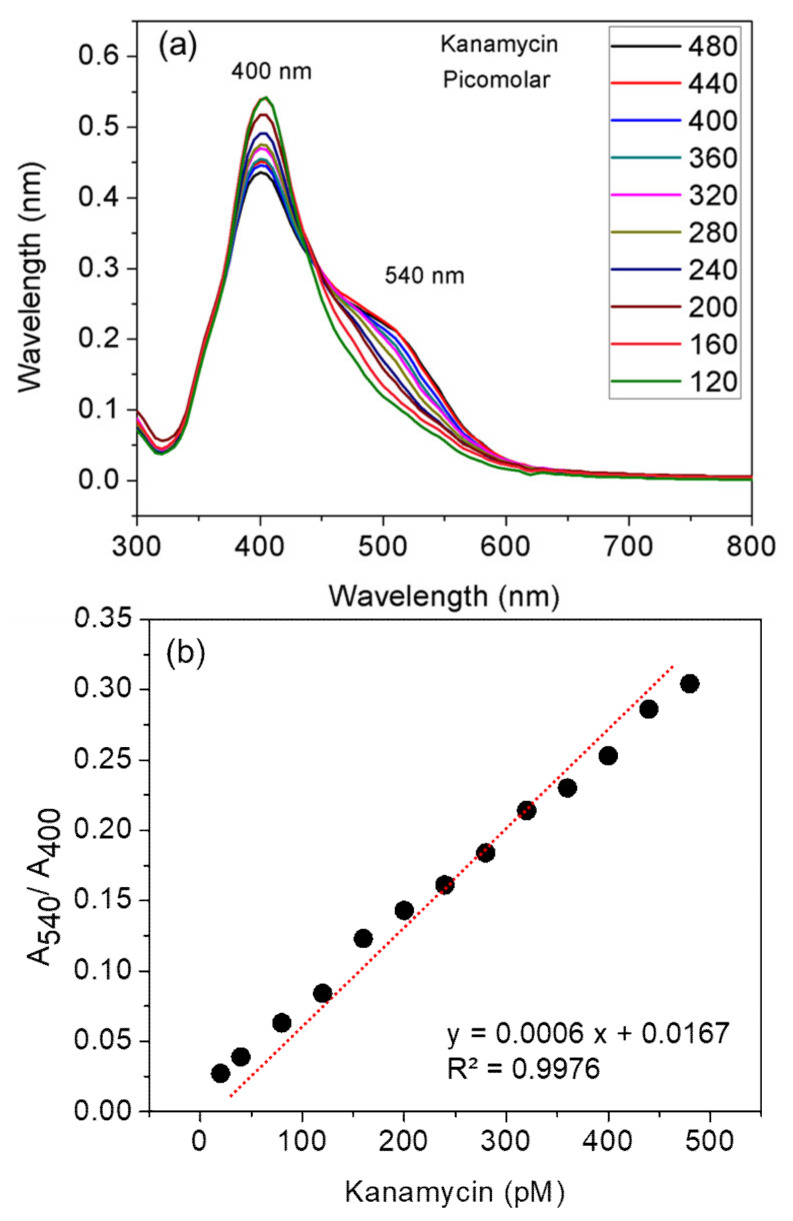
Kanamycin limit of detection analyzed using UV–visible spectroscopy and ratiometric results. Varying concentrations of kanamycin in water were added to chlortetracycline-coated silver nanoparticles in a final volume of 1 mL in the cuvette. (**a**) The UV–vis spectra obtained for each concentration of kanamycin and (**b**) the linear range observed for ratiometric measurement (A_540_/A_400_). The values represent the average of triplicates of each set.

For the quantitative detection of kanamycin, the UV-vis spectra of different concentrations of kanamycin were measured in the range of 120–480 pM (Figure 4a). When the kanamycin concentration reached 480 pM, the relative absorbance intensity at 540 nm was stabilized (Figure 4a). The UV–vis spectra labeled with red and black lines correspond to 440 and 480 pM kanamycin, respectively.

Then, the absorbance at 400 and 540 nm was recorded. The ratio of the absorbance at these two selected wavelengths was calculated and plotted against the concentration of kanamycin. As shown in Figure 4b, the value of A_540_/A_400_ increased linearly with the increase in kanamycin concentration, and the color changed from yellow to orange or red. Figure 4b shows a linear response of ratiometric results over the picomolar concentrations of kanamycin (120–480 nM), wherein the linear equation was *y* = 0.0006*x* + 0.0167, the R^2^ was 0.997, and the calculated LOD was about 160 pM. According to the maximum residue limits of some AMG antibiotics in water, the LOD value obtained shows that the developed AgNPs probe can be used to sensitively detect both streptomycin [26,27,28] and kanamycin in water [29,30,31,32]. A comparison of the kanamycin LOD value and linear range reported for different methods are listed in Table 2.

### 3.6. Effect of NaCl Concentration

**Figure 5:**
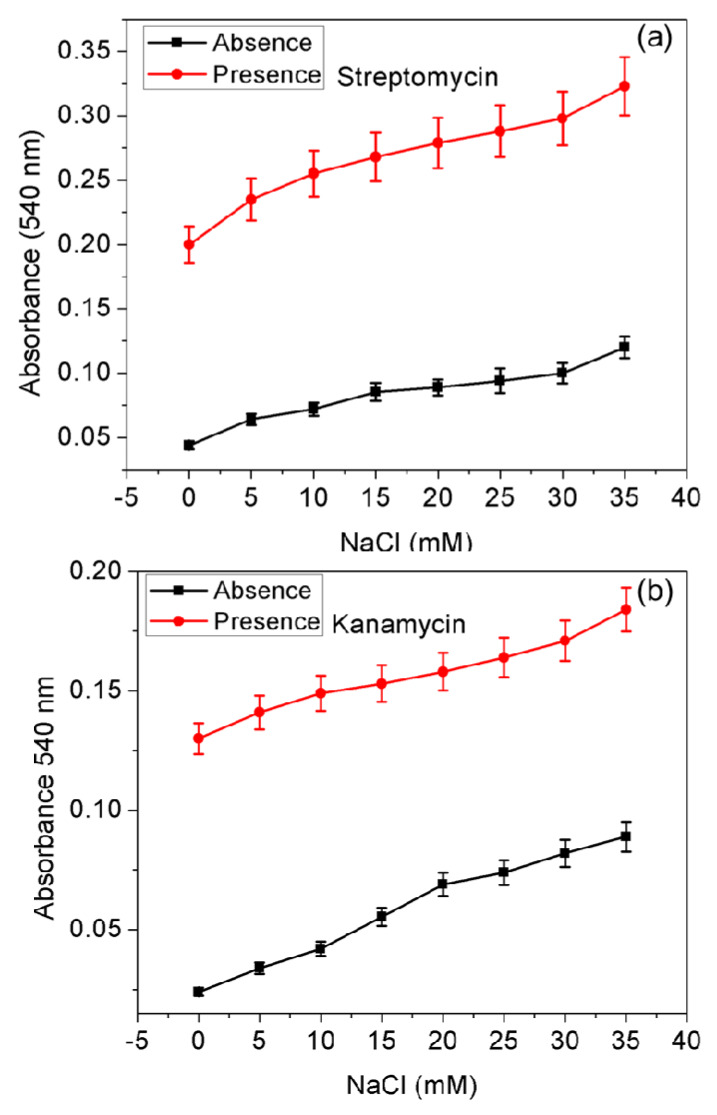
Effect of ionic strength on absorbance intensity of the AgNPs probe recorded at 540 nm in the presence and absence of (**a**) streptomycin and (**b**) kanamycin.

Ionic strength is another important parameter for improving the detection of target analytes, and the dependence of AgNPs absorbance response in the presence of NaCl (final NaCl concentration: 5–35 mM; incubation time 5 min) was investigated. This study is also vital to determine the effect of NaCl for CTC coated AgNP probe against salt-induced aggregation. Aggregation of the AgNPs occurred with the increasing addition of NaCl (Figure 5). Subsequent addition of streptomycin and kanamycin induced the capped-CTC to detach from AgNPs, followed by aggregation depending on the NaCl concentration.

From Figure 5, it can be seen that the AgNPs probe was responsive to NaCl, and increased sensitivity of the AgNPs probe was observed. However, to obtain improved sensitivity, the use of NaCl is not recommended, and NaCl was not used in the other experiments described herein because it may produce false-positive results, as reported elsewhere [42]. The authors clarify that the developed method is already ultrasensitive to AMG antibiotics and suitable for monitoring fresh water samples.

### 3.7. Stability of AgNPs Probe

**Figure 6:**
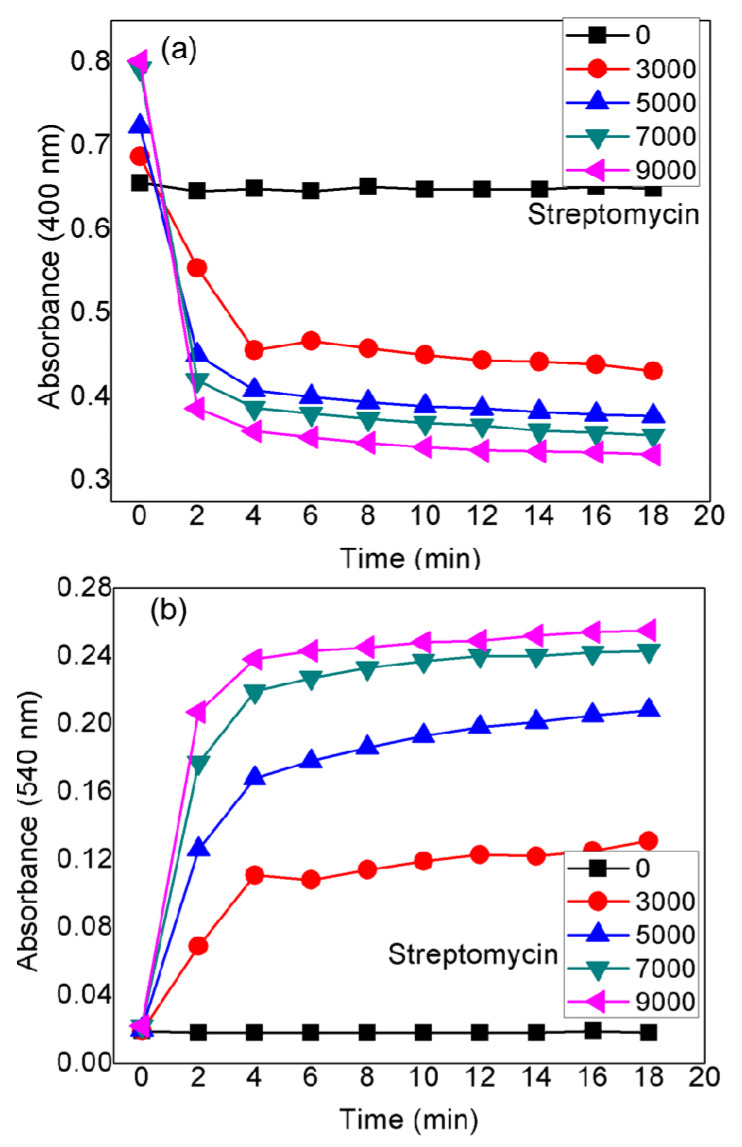
Real-time absorbance response of the AgNPs probe recorded at (**a**) 400 nm and (**b**) 540 nm in the presence of streptomycin at the concentrations indicated.

Figure 6 presents the real-time response recorded for the AgNPs–CTC toward the different concentrations of streptomycin (3000 to 9000 pM) using absorbance data from two different wavelengths, 400 and 540 nm. As seen in Figure 6a, the absorbance intensity at 400 nm decreased rapidly within 2 min for all tested concentrations. Initially, the absorbance intensity decreased promptly to a minimum, and then a further minor decrease was observed, resulting in a more stable sensing platform.

Figure 6b presents a real-time response recorded for the red-shifted wavelength, 540 nm. It shows that the absorbance intensity increased promptly within 2 min and then stabilized as the reaction time was extended. Irrespective of the initial streptomycin concentration, the stable response time for the AgNPs–CTC was found within the window of 5 to 20 min. These results indicate that the absorbance probe could be used at two different wavelengths for the detection of streptomycin in the water samples.

**Figure 7:**
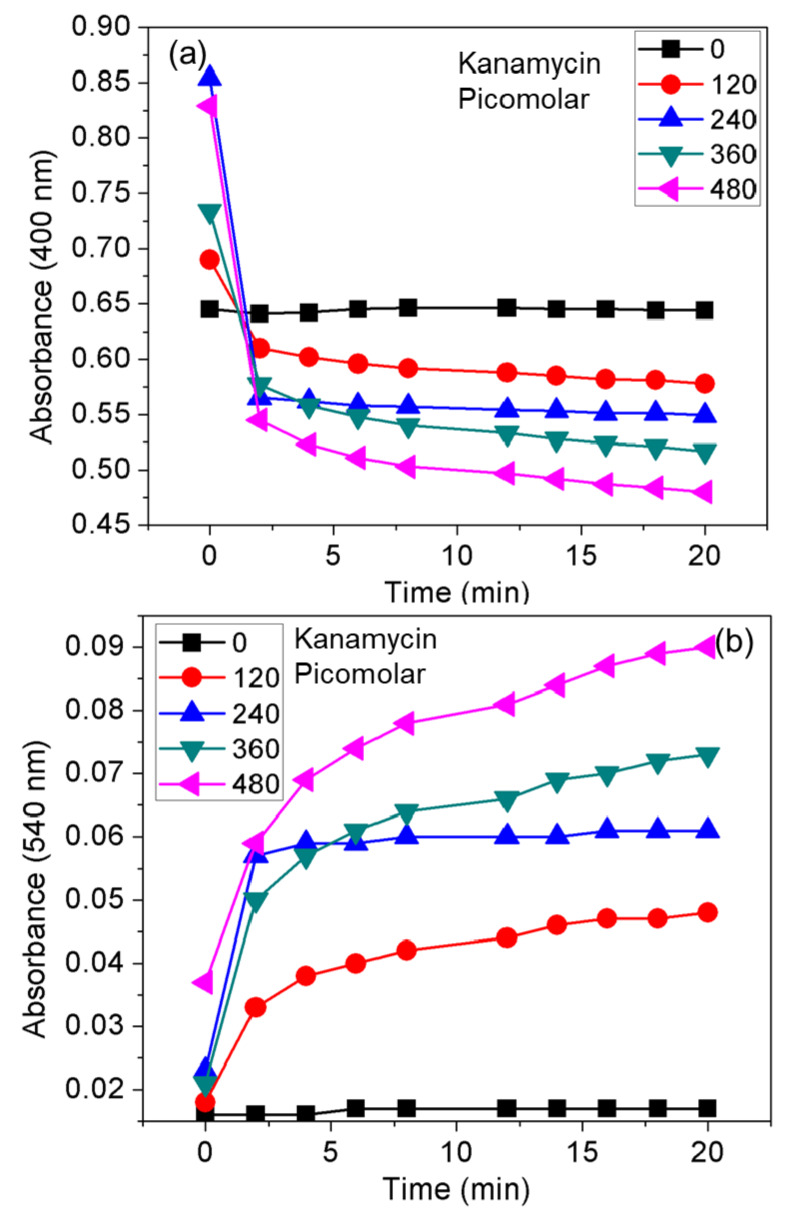
Real-time absorbance response of the AgNPs probe recorded at (**a**) 400 nm and (**b**) 540 nm in the presence of kanamycin (120, 240, 360, and 480 pM).

The time-dependent absorbance intensity (Figure 7) was also studied by directly adding four different concentrations of kanamycin over the range of 120–480 pM to the solution of AgNPs–CTC. From Figure 7a, the absorbance intensity at 400 nm promptly decreased to a minimum and further decreased gradually, indicating aggregate formation is a rapid process that is completed within 2 min. The opposite trend was observed at 540 nm (Figure 7b).

At all tested concentrations of kanamycin, a stable response for the AgNPs–CTC was observed after an initial increase to the maximum. Such findings indicate the possibility of rapid detection of AMG antibiotics in the water samples within the time frame of 5 to 20 min. In comparison, the colorimetric results, which were highly reproducible and reliable, were visible for a longer period (data not shown). The color of the reaction mixture remained stable for a time frame of minutes to hours, without the formation of agglomeration or precipitation.

## 4. Conclusions

Herein, we have shown that SPR can be used to analyze various AMG antibiotics. For the first time, a CTC-coated AgNPs probe was reported for the rapid colorimetric detection of AMG antibiotics in an aqueous system. The organic ligand-induced aggregation of the AgNPs–CTC probe was found to be a sensitive and cost-effective ratiometric assay for AMG antibiotics. The AMG antibiotics serve not only as target analytes but also as molecular linkers for electrostatic coupling with the AgNPs–CTC probe, giving rise to a noticeable color change from yellow to red that can also be detected by UV–vis spectroscopy. Analysis of streptomycin and kanamycin in water samples was demonstrated, with excellent picomolar-level sensitivity. Thus, this validated ratiometric probe can find analytical applications in the ultrasensitive detection of AMG antibiotics.

## Figures and Tables

**Table 1:** Limit of detection (LOD) for streptomycin using different methods.

Method	Sample	Range (nM)	LOD (nM)	Reference
Silver nanoparticles (NPs)	water	0.05–0.75	0.036	[29]
Immuno-biosensor	milk	31.8–3180	13.03	[30]
Fluorescence	serum	30–2030	47.6	[31]
Competitive enzyme-linked immunosorbent assay (ELISA)	milk	0.31–3180	6.36	[32]
Gold nanoparticles (NPs)	buffer	100–500	86	[33]
Electrochemical	milk	0.15–318	1.59	[34]
Fluorescent apta-sensor	buffer	50–1060	54.5	[35]
Silver nanoparticles (NPs)	water	1–11	2	This method

**Table 2:** Limit of detection (LOD) for kanamycin using different methods.

Method	Sample	Range (nM)	LOD (nM)	Reference
Fluorescent aptasensor	buffer	0.001–1.0	0.002	[36]
Gold NPs	water	20–100	9.21	[37]
Photoelectrochemical	Na_2_SO_4_	0–250	0.2	[38]
Gold NPs	water	1–100	0.75	[39]
Fluorescence	water	0–50	0.437	[40]
Fluorescence resonance	water	100–600	13.5	[41]
Silver NPs	water	0.12–0.48	0.16	This method
